# Single-cell RNA sequencing of adult mouse testes

**DOI:** 10.1038/sdata.2018.192

**Published:** 2018-09-11

**Authors:** Soeren Lukassen, Elisabeth Bosch, Arif B. Ekici, Andreas Winterpacht

**Affiliations:** 1 Institute of Human Genetics, Friedrich-Alexander-Universität Erlangen-Nürnberg, Schwabachanlage 10, 91054 Erlangen, Germany

**Keywords:** Spermatogenesis, Gene expression, Next-generation sequencing, Mouse

## Abstract

Spermatogenesis is an efficient and complex system of continuous cell differentiation. Previous studies investigating the transcriptomes of different cell populations in the testis relied either on sorting cells, cell depletion, or juvenile animals where not all stages of spermatogenesis have been completed. We present single-cell RNA sequencing (scRNA-Seq) data of 2,500 cells from the testes of two 8-week-old C57Bl/6J mice. Our dataset includes all spermatogenic stages from preleptotene to condensing spermatids as well as individual spermatogonia, Sertoli and Leydig cells. The data capture the full continuity of the meiotic and postmeiotic stages of spermatogenesis, and is thus ideally suited for marker discovery, network inference and similar analyses for which temporal ordering of differentiation processes can be exploited. Furthermore, it can serve as a reference for future studies involving single-cell RNA-Seq in mice where spermatogenesis is perturbed.

## Background & Summary

Spermatogenesis, the process in which mature sperm cells are derived from stem cells, has been the focus of extensive research for over a century. During the premeiotic stages, some A-type spermatogonia divide without differentiation to replenish the stem cell pool, while others start differentiating in a series of mitoses marked by incomplete cytokinesis^1^. Differentiated B-type spermatogonia then enter meiosis, a two-step division generating cells with a haploid (1n) genome from diploid (2n) cells. Meiosis I, also known as reduction division, is marked by a lengthy prophase during which homologous chromosomes align, recombination occurs and the X and Y chromosome are silenced^2^. After a relatively short meiosis II, the cells enter the round spermatid stage, during which cytoplasmic and nuclear remodelling is initiated. The round spermatids begin to elongate, forming elongated spermatids, which initiate the transcription of protamines. These proteins are then exchanged for histones, leading to a compaction and general silencing of the nucleus in the successive condensing/condensed spermatid stages^3^.

Previous studies investigating transcriptional dynamics during spermatogenesis predominantly used one of two methods. In many cases, cell populations were sorted according to the expression of protein markers, and sorted cells were subjected to bulk RNA-Seq. This approach is hampered by the availability of specific markers, and thus does not allow a high resolution with regard to the cell populations. Another technique utilizes the testes of juvenile mice at time points when later cell populations have not yet developed^4–6^. In this scenario, the testes are obtained at various postnatal stages and RNA is extracted from total tissue and sequenced^7–9^. While this approach is not based on markers, it leads to noisy data at later time-points. Furthermore, differences between the first and successive waves of spermatogenesis have been described, so it is not clear whether the data obtained in this case can be transferred to adult mice^10^.

**Figure 1:**
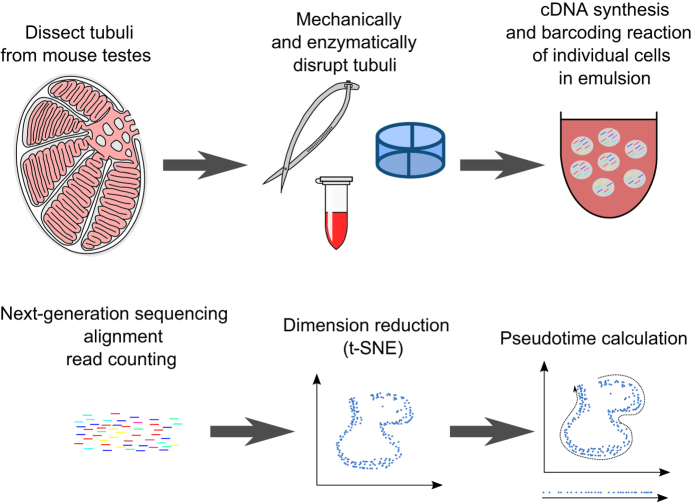
Schematic representation of the workflow used in this study.

To overcome these drawbacks, we decided to generate a scRNA-Seq dataset of testis tissue comprising over 2,500 cells from two eight-week-old C57Bl/6J littermates using the 10X Genomics Chromium platform (Fig. 1). As no selection through marker expression or developmental stage was performed, this dataset provides an unbiased atlas of meiotic and postmeiotic cell populations in the adult mouse testis. The cells can be ordered by pseudotime, allowing any method where temporal ordering is necessary or beneficial, such as stage marker identification or network inference, to be applied. Analyses based on this dataset investigating X and Y expression, pathway activity and transcript-protein-discrepancies have been published^11^. The dataset is thus suited for use by scientists interested in the role of single genes in the testis, as well as those wishing to use it as a reference to compare their transcriptomic data on spermatogenesis in various other mouse models to.

## Methods

These methods are an expanded version of the method section in our related work^11^.

### Animals used

Two eight-week-old wildtype C57Bl/6J mice (Charles River, Wilmington, MA) were used in this study. After sacrificing the mice, the testes were dissected, and single-cell suspensions were prepared as detailed below. As no experiments were performed on live animals, no prior approval was mandated by local law. The study was registered with the Friedrich-Alexander-University Erlangen-Nürnberg and the City of Erlangen (reference: TS-04/12).

### Preparation of cell suspensions

Tissues were prepared as previously described for flow cytometry analysis of testicular cells^12^. The testes were collected in ice cold PBS and the tunica albuginea was removed using forceps. 10 mg of tissue (approximately 1/10^th^ of total testis weight) was collected in Protein LoBind tubes (Eppendorf). The tissue was minced in 200 μl of cold digestion medium (1 mg/ml collagenase/dispase, 1 mg/ml hyaluronidase and 1 mg/ml DNaseI in DMEM/F12) using McPherson-Vannas scissors. 800 μl of digestion medium was added and the reaction was incubated for 20 min at 37 °C under slow rotation. Every 5 min the solution was pipetted up and down using wide bore tips. The cell suspension was then slowly filtered through a 40 μm cell strainer to obtain single cells and to minimize the number of somatic cells. The cells were pelleted by centrifugation (400 g, 10 min, 4 °C), the supernatant was discarded and the pellet was resuspended in 1 ml ice cold PBS. Cell numbers and viability were assessed by Trypan blue staining and counting in a Neubauer improved counting chamber.

### Single-cell library preparation and sequencing

Libraries were prepared using the Chromium controller (10X Genomics, Pleasanton, CA) in conjunction with the single-cell 3’ v2 kit. Briefly, the cell suspensions were diluted in nuclease-free water according to manufacturer instructions to achieve a targeted cell count of 1,000–2,000. cDNA synthesis, barcoding, and library preparation were then carried out according to the manufacturers’ instructions.

The libraries were sequenced on an Illumina HiSeq 2500 (Illumina, San Diego) with a read length of 26 bp for read 1 (cell barcode and unique molecule identifier (UMI)), 8 bp i7 index read (sample barcode), and 98 bp for read 2 (actual RNA read). Reads were first sequenced in the rapid run mode, allowing for fine-tuning of sample ratios in the following high-output run. Combining the data from both flow cells yielded approximately 200 M reads per mouse.

### Primary single-cell data analysis

The reads were demultiplexed by using cellranger (2.0.0, 10X Genomics) mkfastq in conjunction with bcl2fastq (2.17.1.14, Illumina).

As transposable elements (TEs) have been described to be differentially regulated during spermatogenesis, we decided to include these in our analysis. To this end, we obtained the fasta sequences of transposable elements in the mouse from RepBase 17.04 and generated a custom reference genome. In a first step, we downloaded a repeat-masked version of the GRCm38 genome from Ensembl release 89 and added the TEs as individual chromosomes. We then updated the annotation gtf file to include the position of the transposable elements. This strategy was necessary to avoid the exclusion of transposon-derived reads due to their multi-mapping nature.

The reads were then aligned to the reference genome, filtered, and counted using the cellranger count command. As two libraries were generated (from the rapid run as well as the high-output run), a .mro file combining both flow cells was written as detailed in the cellranger documentation. The data for the two mice was then combined through cellranger aggr. This step includes downsampling of reads to achieve the same mean number of reads per cell across all samples. This resulted in 8.7% of reads from mouse 2 being discarded, leading to an average of 148,104 reads per cell. Cellranger aggr was further used to generate an initial secondary analysis (t-distributed stochastic neighbor embedding (t-SNE), graphbased clustering, K-means clustering for K=2–10). 42 was used as random seed.

The t-SNE projection (standard settings; perplexity: 30, theta: 0.5, 1,000 iterations maximum, learning rate and momentum reduction at 250, 10 components from principal component analysis (PCA/IRLB) as input) arranged the cells in a continuous succession, indicating the possibility of a pseudotime analysis (see below).

For K-means clustering, the largest number of clusters that did not place a single cell as cluster (K=9) was chosen. The cell type of the individual clusters was assigned through known markers, which confirmed the distinct identities of the clusters identified (see below).

### Quality control

Common quality control measures for single-cell RNA-Seq (gene count per cell, UMI count per cell, percent of mitochondrial transcripts) were calculated using the Seurat R package^13^ (version 2.2). The analyses were performed for the combined replicates and for each mouse individually. The corresponding code used is available online (see Code availability).

### Pseudotime analysis

Pseudotime analysis was performed using the R packages monocle^14–16^ (version 2.6.1) and scrat^17–19^ (version 1.0.0). For analysis with monocle, filtered gene/barcode matrices containing both replicates were loaded, using a lower detection limit of 0.5 and a minimum mean expression per gene of 0.1. To further reduce the number of genes used, a threshold of 5% was introduced for the number of cells in which genes for downstream analysis were expressed. Genes passing this filtering step were subjected to differential expression calculation, with only the top 1,000 genes (by q-value) used for pseudotime calculation. The exact code used can be accessed below.

For pseudotime analysis using scrat, count processing and feature centralization were set to true, while cell cycle correction and sample normalization were turned off. Cell cycle correction was not deemed necessary, as only few cells (~20) were expected to be undergoing mitotic divisions. Pseudotime calculation itself was performed with 20 waypoints, 20 iterations, K=30, I=5, and an appropriate initiator sample (cell number 924) visually chosen from the t-SNE plot. As with the script used for monocle, access is described below.

Correlation calculations were performed on the pseudotime ranks derived for each cell using both methods. As the correlation coefficient (Spearmans’ R) was determined to be 0.98, no method was clearly superior.

### Cell type assignment

Vast literature research identified 233 already published markers for spermatogenesis, identified through detection on either RNA or protein levels or both (Lukassen *et al.*, 2018, Supplementary data table 2^11^). Of these genes, 224 were annotated in the present dataset. In order to assign the different cell populations present in the testis to the clusters identified through K-means clustering, marker gene expression was plotted along the pseudotime axis and against the different clusters using the Seurat package. To include only genes with relevant expression levels in the dataset, genes expressed in at least 3 cells with a mean expression of 0.1 over all cells were included, resulting in 214 markers (Lukassen *et al*., 2018, Supplementary Figure S3^11^). Marker genes with cluster-specific expression were then used to define the corresponding cell type (Lukassen *et al.*, 2018, Fig. 1 and Supplementary data table 3^11^).

### Code availability

The R code used in the analysis of the single-cell RNA-Seq data is available on GitHub (https://github.com/slukassen/SCS_testis/). The repository includes a session info file with information on the package versions used (Data Citation 1).

## Data Records

The sequencing data from this study has been uploaded to GEO and SRA (Data Citation 2 and Data Citation 3). This includes three raw .fastq files for each mouse (*I1.fastq.gz: index read; *R1.fastq.gz: cell barcode and UMI; *R2.fastq.gz: RNA read). Furthermore, an expression matrix in matrix market exchange format (*.mtx) is included, with columns corresponding to cells and rows to genes. The identifiers for the columns and rows are included as separate files (barcodes.tsv and genes.tsv). These processed files correspond to the output produced by the cellranger pipeline and placed in “runfolder/outs/filtered_gene_barcode_matrices_mex/genome/”. In addition, a Supplementary table is supplied with this paper that contains information on each cell: Barcode, Replicate ID, t-SNE position, UMI count, gene count, mitochondrial transcript levels, Cluster ID from K-Means clustering (K=9), Cell Type, and pseudotime information calculated using monocle and scrat are included (Data Citation 1). A second Supplementary table lists expression information for each expressed gene in all cell populations (Data Citation 1).

## Technical Validation

**Figure 2:**
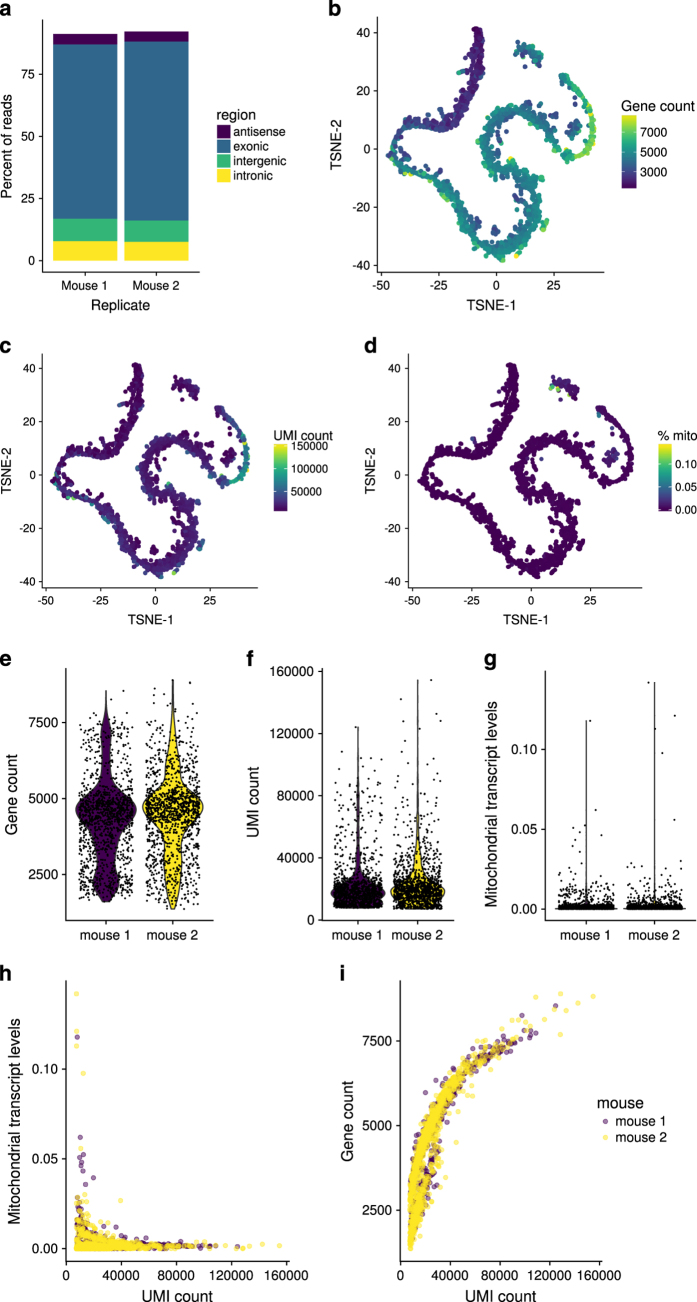
Quality control plots for single-cell RNA-Seq. (**a**) Mapping positions of confidently mapped reads in the two replicates. (**b**) The gene count mapped onto the t-SNE projection. (**c**) The UMI count mapped onto the t-SNE projection. (**d**) The proportion of mitochondrial genes mapped onto the t-SNE projection. (**e**) Violin plot showing the distribution of gene counts, split by replicates (mouse 1: purple, mouse 2: yellow). (**f**) Violin plot showing the distribution of UMI counts, split by replicates. (**g**) Violin plot showing the distribution of mitochondrial transcript levels, split by replicates. (**h**) Scatterplot showing the correlation of mitochondrial transcript levels and UMI count. (**i**) Scatterplot showing the correlation of gene count and UMI count.

To assess the quality of the cDNA synthesis and barcoding steps, especially with regard to DNA contamination, the mapping location of aligned reads was assessed. As expected, the majority of confidently aligned reads mapped to exonic regions, with little background in introns, intergenic regions, or antisense to exons (Fig. 2a). As non-exonic binding could be attributed to actual transcripts missing from the annotation as well as to DNA contamination this indicated successful cDNA generation and sequencing. The two biological replicates were virtually indistinguishable in terms of mapping, further supporting the quality of the data.

After normalization (downsampling of the more deeply sequenced replicate to achieve equal read-depth per cell), the cells of both animals were pooled *in silico* for downstream analyses. In the t-SNE projection, no difference could be observed between the replicates, even though no alignment was performed to regress out between-sample heterogeneity. Furthermore, the numbers of spermatocytes, round spermatids, elongating spermatids, and condensed/condensing spermatids did not vary by more than 25%. Even clusters with less than 10 cells (spermatogonia, Sertoli cells, Leydig cells) were present in both replicates.

The expression of published stage markers was in agreement with both the cell type inferred here and the timing of differentiation. This was both true at the individual marker levels and the general expression patterns observed in previous studies employing cell sorting and bulk RNA-Seq. In some cases (*Kit* and *Pou5f1*) where a difference was observed between published protein expression data and the transcription profile observed in this study, this could be attributed to previously described non-coding isoforms^20,21^.

As different cell populations in the testis have a highly variable RNA content, with the highest levels observed in pachytene spermatocytes, an enrichment of both the gene count and UMI count was expected in this population^22^. This was indeed observed in the data (Fig. 2b and c). Previous bulk RNA-sequencing data of testicular cell populations indicates a higher number of expressed genes (e.g. Soumillon *et al.*: roughly 14,000 genes with an expression >1 FPKM)^22^. Their data shows many of these genes to be very weakly expressed, with a mean of less than 0.2 FPKM^22^. At 150,000 reads per cell, a 1 kb transcript would have to be expressed at 4.7 FPKM to be detected in more than half of all cases. As this would put the detectable transcripts in the range of the 75^th^ to 80^th^ percentile of published data, the distribution of gene count agrees with our expectations. Previously published data on the RNA content of different cells in the mouse testis report a roughly 6fold difference for RNA-rich and RNA-poor populations^22^. More than 80% of the cells assayed here fall within this range of variability. The percentage of mitochondrial transcripts is a frequently used indicator of apoptotic cell populations. In our data, only nine cells surpassed the commonly used threshold of 5% mitotic transcripts. As these cells were found in the population identified as Sertoli cells, which provide nutritional support to the surrounding germ cells^23^, the increased mitochondrial content may be due to an increased metabolism rather than apoptosis (Fig. 2d). The distribution of gene counts, UMI counts, and mitochondrial transcript levels was indistinguishable between both biological replicates (Fig. 2e–g). While a two-sample, independent, unequal-variance t-test on the gene count in both replicates did yield a p-value of 0.0012, the difference in means was 172 (68–277 95% confidence interval), or about 4% of the mean. This indicates that a detectable difference was present, as would be expected for biological replicates and large numbers of cells, but the overall effect strength was negligible. Both the comparison of the UMI count and the percentage of mitochondrial transcripts yielded a p-value that was larger than 0.1 (0.19 and 0.55, respectively). Cells with high levels of mitochondrial transcripts tended to have a lower total UMI count, which could point toward some apoptotic cells being present in the dataset (Fig. 2h). The absolute values for the correlation coefficients were below 0.1 for both mice (0.027 and 0.084). As expected, there was a positive correlation between the UMI count and the number of detected genes (Spearman’s ρ of 0.90 and 0.92 for mouse 1 and 2, respectively, p<2.2E-16 for Spearman’s test with asymptotic t-approximation), which flattened toward high UMI counts (Fig. 2i). Again, these correlations did not differ between both mice.

## Usage Notes

The raw data in .fastq format (Data Citation 3) can be used as input for the cellranger pipeline or similar tools. The gene-barcode matrices supplied can be placed in a folder that is passed to the Read10X function of the Seurat R package. The count matrix can then be analysed by a variety of tools, such as monocle, Scrat, Seurat, or SCENIC. Examples for some basic analysis scripts used for generating the data presented here are available online (see the Code availability section).

As the RNA content of individual cell populations in the mouse testis varies greatly^22^, it is important to select an appropriate normalization strategy. When comparing the data to bulk RNA-Seq from sorted cells, normalization to the UMI count per cell should be performed, while this will lead to incorrect results in comparisons with techniques such as *in-situ* hybridization.

## Additional information

**How to cite this article**: Lukassen, S. *et al*. Single-cell RNA sequencing of adult mouse testes. *Sci. Data* 5:180192 doi: 10.1038/sdata.2018.192 (2018).

**Publisher’s note**: Springer Nature remains neutral with regard to jurisdictional claims in published maps and institutional affiliations.

## Supplementary Material
